# Operationalising Regional Cooperation for Infectious Disease Control: A Scoping Review of Regional Disease Control Bodies and Networks

**DOI:** 10.34172/ijhpm.2021.176

**Published:** 2021-12-26

**Authors:** Anna Durrance-Bagale, Manar Marzouk, Sunanda Agarwal, Aparna Ananthakrishnan, Sarah Gan, Michiko Hayashi, Beth Jacob-Chow, Koh Jiayun, Lam Sze Tung, Hala Mkhallalati, Sanjida Newaz, Maryam Omar, Manit Sittimart, Mengieng Ung, Yang Yuze, Hsu Li Yang, Natasha Howard

**Affiliations:** ^1^National University of Singapore, Saw Swee Hock School of Public Health, Singapore, Singapore.; ^2^London School of Hygiene and Tropical Medicine, London, UK.; ^3^Stanford Distinguished Careers Institute, Stanford, CA, USA.; ^4^Health Intervention and Technology Assessment Program, Ministry of Public Health, Nonthaburi, Thailand.; ^5^Department of Community Health Sciences, Rady Faculty of Health Sciences, University of Manitoba, Winnipeg, MB, Canada.; ^6^Chelsea and Westminster Hospital NHS Foundation Trust, London, UK.; ^7^Lee Kuan Yew Centre for Innovative Cities, Singapore University of Technology and Design, Singapore, Singapore.

**Keywords:** Infectious Disease, Cooperation, Networks, Regional Organisations, Southeast Asia

## Abstract

**Background:** The rapid spread of the coronavirus disease 2019 (COVID-19) pandemic demonstrates the value of regional cooperation in infectious disease prevention and control. We explored the literature on regional infectious disease control bodies, to identify lessons, barriers and enablers to inform operationalisation of a regional infectious disease control body or network in southeast Asia.

**Methods:** We conducted a scoping review to examine existing literature on regional infectious disease control bodies and networks, and to identify lessons that can be learned that will be useful for operationalisation of a regional infectious disease control body such as the Association of Southeast Asian Nations (ASEAN) Center for Public Health Emergency and Emerging Diseases.

**Results:** Of the 57 articles included, 53 (93%) were in English, with two (3%) in Spanish and one (2%) each in Dutch and French. Most were commentaries or review articles describing programme initiatives. Sixteen (28%) publications focused on organisations in the Asian continent, with 14 (25%) focused on Africa, and 14 (25%) primarily focused on the European region. Key lessons focused on organisational factors, diagnosis and detection, human resources, communication, accreditation, funding, and sustainability. Enablers and constraints were consistent across regions/ organisations. A clear understanding of the regional context, budgets, cultural or language issues, staffing capacity and governmental priorities, is pivotal. An initial workshop inclusive of the various bodies involved in the design, implementation, monitoring or evaluation of programmes is essential. Clear governance structure, with individual responsibilities clear from the beginning, will reduce friction. Secure, long-term funding is also a key aspect of the success of any programme.

**Conclusion:** Operationalisation of regional infectious disease bodies and networks is complicated, but with extensive groundwork, and focus on organisational factors, diagnosis and detection, human resources, communication, accreditation, funding, and sustainability, it is achievable. Ways to promote success are to include as many stakeholders as possible from the beginning, to ensure that context-specific factors are considered, and to encourage employees through capacity building and mentoring, to ensure they feel valued and reduce staff turnover.

## Background

The rapid spread of the novel coronavirus disease 2019 (COVID-19) pandemic, beginning in late 2019 and now affecting almost all countries globally^1,2^ demonstrates the value of regional cooperation in infectious disease prevention and control. Infectious diseases do not respect national borders and countries are only as safe as their neighbours during pandemics. Regional responses to COVID-19 have been marked by individual country-led efforts and minimal regional collaboration.^3^ Prevention and control of diseases with epidemic potential demand emergency responses and flexibility at national and regional levels. Control of communicable and infectious diseases is a global public good that affects everyone, as illustrated by the COVID-19 pandemic and the obvious need for effective vaccination programmes around the world.^4^ Collective action that reduces the prevalence of an infectious disease in one country will benefit other countries, as the potential for spread is reduced.^5^ For this, regional and national institutions that encourage and facilitate cooperation between actors, within and across borders, are essential.^6^

Consolidation of regional disease control efforts is motivated on the premise of economies of scale and scope, such as rationalisation of administrative burdens, human resources, and funding, with additional benefits of having a coherent infectious disease control agenda instead of fragmented and siloed programmes and activities, and the practical benefit of being able to monitor potential disease spread across borders. As countries ease lockdowns and relax border controls, practical concerns drive the need to develop transnational mechanisms for continued infectious disease surveillance and response, especially as migration, trade, and supply chains expand or restart across regions. In contrast to the European Union (EU) and African Union, there is no equivalent regionalisation of disease control in Southeast Asia (SEA), our region of focus, although SEA has been the epicentre of several public health crises, such as severe acute respiratory syndrome (SARS) and avian influenza in 2003.^7,8^ Instead of leadership by a regional cooperation body such as Association of Southeast Asian Nations (ASEAN), a patchwork of independent initiatives fulfils aspects of this role, informed by differing agendas and timeframes that lead to duplication of objectives and lack of continuity or synergy.^9^ After SARS in April 2003, ASEAN Health Ministers agreed on joint action for “the sharing of experience and best practices between countries,” which spanned knowledge exchange and harmonisation of travel procedures and records. However, these coordination efforts were limited to one of 46 divisions in the ASEAN corporate structure, and the Charter’s main activities remained focused on regional economic activity and integration.^9^

## Aim and Objectives

This review explored the literature on operationalising regional infectious disease control bodies and agreements. Objectives were to: (*i*) summarise the scope (ie, extent, nature, distribution) of existing literature on regional infectious disease control bodies and their operationalisation; (*ii*) summarise examples; and (*iii*) identify lessons, barriers and enablers from this literature to inform operationalisation of any new regional infectious disease control body in the SEA region.

## Methods

###  Study Design

We conducted a scoping review using Arksey and O’Malley’s six-stage scoping framework with Levac and colleagues’ 2010 revisions and Khalil and colleagues’ 2016 refinements.^10-14^ Scoping reviews are ‘particularly useful when a body of literature has not yet been comprehensively reviewed, or exhibits a complex or heterogeneous nature not amenable to a more precise systematic review.’^12^ Table 1 shows our working definitions, which were refined as the study progressed.

**Table 1:** Definitions Used in This Study

**ASEAN**	Association of Southeast Asian Nations, a regional grouping promoting economic, political, and security cooperation among its ten members: Brunei, Cambodia, Indonesia, Laos, Malaysia, Myanmar, the Philippines, Singapore, Thailand, Viet Nam^15^
**Disease control**	The reduction of disease incidence, prevalence, morbidity or mortality as a result of deliberate efforts^16^
**Disease prevention**	Specific, population-based and individual-based interventions for primary and secondary (early detection) prevention, aiming to minimise the burden of diseases and associated risk factors^17^
**Operationalise**	Functioning, viable, practicable, workable, fit for purpose, initiate, realise, implement
**Regional bloc**	Our definition of a regional bloc is a group of countries based in a region that have similar aims and interests and that act together over some issues. Examples include the African Union, Association of Southeast Asian Nations, Arab League, Caribbean Community, Council of Europe, Eurasian Economic Union, European Union, South Asian Association for Regional Cooperation, Asian-African Legal Consultative Organization, Union for the Mediterranean, Union of South American Nations, West African Health Organisation

Abbreviation: ASEAN, Association of Southeast Asian Nations.

###  Stage 1. Defining the Research Question

 We specified two research questions:

1. ‘What is the scope (ie, extent, nature, distribution) and main findings of existing literature on regional infectious disease control bodies and how they work?’
2. ‘What lessons can be learned from these experiences that will be useful for an ASEAN disease control body?’

###  Stage 2. Identifying Relevant Studies

 To increase breadth and comprehensiveness, we searched relevant electronic databases and websites (Table 2).

**Table 2:** Electronic Databases and Websites

**Databases**	**Number of Sources**
Medline (Ovid)	3475
Global health (Ovid)	2292
EMBASE (Ovid)	4901
Web of Science	4949
EconLit	140
Eldis	0
Total	15 757
Number of duplicates	6346
After duplicates removed in Endnote	9411
After duplicates removed in Covidence	9395
**Grand Total**	**9395**

 First, we systematically searched published literature in six databases, including a grey literature database. Second, we purposively searched selected websites, including Google, to locate government documents. Finally, we purposively searched the reference lists of all included sources. We used the terms and related terminology for ‘regional’ AND ‘disease control body’ AND ‘operationalisation’ adapted to the subject headings for each database. For example, our Medline search syntax was: 1. (Region* or international or continent*); 2. ((Body or bodies or organi#ation* or centre or center or entity or entities or agreement* or co-opera* or network* or partner* or collaborat* or co-ordinat*) adj5 (disease control or health protection or health response* or disease prevention or public health or surveillance or emergenc* or emerging disease* or infectious or epidemic* or pandemic* or outbreak*)); 3. (Implement* or operation* or run* or function* or establish* or governance or viab* or practic* or initiat*); 4. 1 and 2; and 5. 3 and 4. Similar search terms were used in Google and selected websites.

###  Stage 3. Selecting Studies

We established eligibility criteria via an iterative process, agreeing initial criteria based on the research question (Table 3). Outcomes were restricted to descriptions of evaluation approaches or methods implemented. Source types were restricted to academic and technical literature. All languages were included for documents that had an English abstract. All study designs, interventions, and participants (eg, health-workers, expert panels, service-users) were considered.

**Table 3:** Full Eligibility Criteria

**Criteria**	**Included**	**Excluded**
1. Context	Regional bloc (eg, ASEAN, EU, AFR, PAHO) or group of at least 3 countries	Other settings (eg, subnational, national, bilateral, global)
2. Topic	Regional cooperative body to improve human health (eg, centre for disease control)	Unrelated to human health Unrelated to a regional health organisation, network, or agreement between at least 3 countries
3. Outcomes	Describes set-up or operationalisation/implementation experience, organisational structures, management, purpose, method, or lesson	Other outcomes not related to organisational development and inner workings
4. Source type	Primary research articles Commentaries/editorials/reviews Conference abstracts Books/chapters Organisational reports (eg, government, non-governmental organisations) Government documents Policy briefs	Non-text, eg, audio/video reports Conference abstracts covering the same material as an available publication Social media, blogs, news articles
5. Time-period	Any	NA
6. Language	Any for which an English abstract is available	Sources for which no English abstract is accessible or in a language for which study authors have no proficiency
7. Study design	Any	NA
8. Participants	Any	NA

Abbreviations: ASEAN, Association of Southeast Asian Nations; AFR, African Region; EU, European Union; PAHO, Pan American Health Organization; NA, not available.

**Figure 1:**
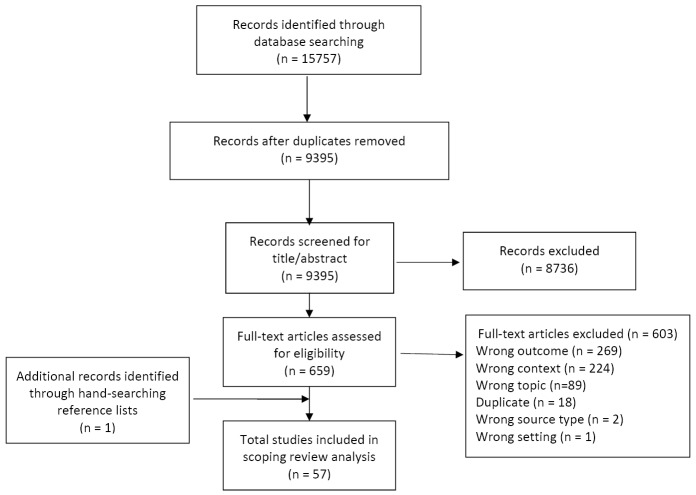
PRISMA Flow Diagram. Abbreviation: PRISMA, Preferred Reporting Items for Systematic Reviews and Meta-Analyses.

First, we identified documents in databases and websites. Second, we removed duplicates using the reference manager EndNote before importing the references into Covidence and removing any further duplicates. Third, we screened titles and abstracts against eligibility criteria to remove irrelevant sources using Covidence software. Fourth, we screened remaining full texts against eligibility criteria to remove ineligible documents. Fifth, we identified any further documents from reference lists of included studies and included them if eligible. This provided our total number of documents included (Figure 1).

###  Stage 4. Charting Data 

We extracted data to an Excel sheet using the following headings: (*i*) source identifiers, ie, publication year, lead author, source type (eg, article, conference abstract/presentation, report); (*ii*) source characteristics, ie, region, study aim, study design, methods (eg, participant characteristics, data collection, analysis); and (*iii*) findings, ie, body type, role, activities, structure, successes, challenges, lessons.

###  Stage 5. Collating, Analysing and Reporting Results

First, we summarised the number of sources by publication year, source type (eg, article, report), distribution (ie, publication language), region involved, nature (ie, study aim, study design, methods), body details (name, type, purpose, reason for initiation, leadership/management, structure/approach) and successes, challenges and useful lessons from the body’s initiation. Second, we identified and summarised regional cases. Third, we analysed data on potential lessons thematically – using inductive coding as described by Braun and Clarke^18^ – guided by research objectives and discussed implications for policy, practice, and future research.

## Results

###  Scope of the Literature 

**Figure 2:**
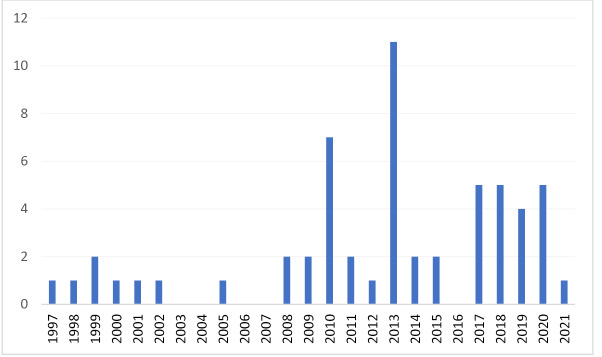
Number of Publications by Year.

*Extent.* Figure 1 shows the Preferred Reporting Items for Systematic Reviews and Meta-Analyses (PRISMA) diagram for the 57 literature sources included of 9395 identified, 56 from databases and 1 from reference lists. Figure 2 shows numbers by publication year, starting with 1 in 1997 with no major increases until 2010 and a peak in 2013. Sources included in the analysis are tabulated in Supplementary file 1.

*Nature*. Most publications were in English (53; 93%), with two (3%) in Spanish and one (2%) each in Dutch and French.^19-22^ Most (48 [83%]) were commentaries or review articles describing programme initiatives, with four (7%) abstracts, three (5%) reports, one (2%) editorial and one (2%) book chapter.

*Distribution*. Regionally, 16 (28%) publications focused on organisations in the Asian continent, 14 (25%) focused on Africa, 14 (25%) discussed organisations with a main focus on the European region, eight (14%) on the Americas, two (3%) on the Pacific or Western Pacific regions, and one (2%) each on Africa/Americas, Africa/Asia and multiregional (Americas/Africa/Europe/Asia).

###  Lessons Learned

Table 4 provides overall lessons learned from included sources. These can be grouped into organisational factors, effective networks, programming, diagnosis and detection, human resources, communication, and sustainability and funding. Enablers and constraints were consistent across regions and organisations. A clear understanding of the regional context, in terms of budgets, cultural or language issues, staffing capacity and governmental priorities is necessary for any organisation. Forming an organisation or initiative with an initial workshop inclusive of the various bodies and staff representatives that will be involved in the design, implementation, monitoring or evaluation of programmes is essential for success. A clear and sensible governance structure, with individual responsibilities clearly delineated from the beginning, will help to reduce any friction or confusion that may arise later in the process. Whether long-term funding is secure and the source of this funding, from national governments, non-governmental organisations (NGOs), or global bodies like the World Health Organization (WHO), is also a key aspect of the success of any programme. Dependency on external funding can cause initiatives to fail when funding bodies such as NGOs pull out of a country, and so securing funding internally is the best option if possible.

**Table 4:** Key Lessons

**Focus**	**Enablers**	**Barriers **	**Lessons**
Organisational factors	Understanding of the context Information technology systems Effective surveillance systems	Regional/contextual context (eg, refugee and migrant populations) Instability and political changes Lack of data so lack of awareness of the existing situation Inter-country differences, including the structure of the surveillance systems Lack of awareness among policy-makers Recognition of cultural and political factors Lack of trust and transparency (eg, some countries may not report outbreaks) Insufficient capacity to plan, mobilise and implement control strategies Lack of political will Requirement to comply with local regulations	Start programme building with a workshop involving representatives from public health, clinicians, laboratory staff, academia, ministries of health, public and private hospitals to understand relevant contexts Clear governance structure is crucial: all members need to outline their own specific immediate objectives that would help to achieve the overall goal Common goals need to be agreed and leveraged through the national structures Have an inclusive One Health focus: animal, human and environmental The general data protection regulation legislation in the EU limits data sharing, even when this may be useful. Certain countries may need to improve their compliance with data handling and reporting laws so they can be included in a body Countries in the network should select microbiological laboratories to serve as sentinel laboratories, and one should be designated a National Reference Laboratory
Networks	Establishing collaborative platforms Public-private partnerships Relationship/partnership building	Lack of coordination between health and other sectors Lack of trust between organisations/partners Lack of public-private partnerships	Cross-country networks are effective for supporting peer-to-peer learning, and have the potential to generate efficiencies in responding to disease outbreaks Collaboration that brings different specialties and types of provider together allows a sense of ownership at both national and local levels to develop and increase sustainability Identify focal people in each country involved in an organisation Collaboration with other established regional entities helps to exchange experiences and lessons Collaboration with other non-health sectors including ministries of education, agriculture and environment
Programming	Programme management Introduction of vaccination programmes Identification of priority diseases Strategy setting Economic evaluations of programmes	Lack of strategic planning	Regional peer audit mechanism is one effective tool for tracking the performance of programmes at each partnering country, and for exchanging knowledge Economic evaluations should become routine, to inform decision-making and prioritising public health interventions Programmes should have enough funding and resources to address social determinants of health Combining research with policy is a useful tool to build strong advocacy points in international debates
Diagnosis and detection	Sufficient laboratory capacity Standardisation/harmonisation	Lack of laboratory capacity and necessary equipment Lack of infrastructure (eg, no hospitals/clinics in rural areas) Lack of cold chain facilities Lack of sufficient/poor quality data to allow planning	Centralise the laboratory network under one organisation to increase cost efficiency and standardisation of procedures Set up an online data repository that all members of the network can access, to upload and download relevant data
Human resources	Sufficient, well qualified staff Staff retention Capacity building/training	Lack of trained, qualified staff especially in the field of microbiology Lack of mentorship programmes to follow up on trained staff High staff turnover Cultural issues (eg, ensuring women see women) Lack of cross-disciplinary working: staff work in specialty silos	Build the human and technical capacity of partnering organisations so they all have similar skill levels Provide staff regular opportunities to feedback on policies and programmes Discuss human resources issues with staff to encourage buy-in Have a mentoring programme for junior staff
Communication	Dissemination of bulletins to keep health workers, donors, governments and the public informed of progress Health-related publications Designing national guidelines	Different languages and dialects Difficulty of disseminating concepts to people with little education Low literacy rate in some regions	Have standard operating procedures written and accessible on a website for anyone to access at any time Implement public awareness programmes for the public and health workers Implement a ‘Plain English’ (or relevant language) initiative to make all literature comprehensible Provide regular updates on network activities that are accessible to all
Sustainability and funding	Funding from governments rather than NGOs	Competing priorities for funding Dependency on external funding Inability to secure funding for long-term programmes Lack of government/political will to provide funding Lack of clear plans to use available funding and how to allocate it Underfunded healthcare systems, so infectious disease is not a priority	Ensure funding is in place before beginning programmes to ensure sustainability and longevity of programmes Secure funding from internal organisations rather than NGOs/external funding bodies Invest in funding to improve the laboratory capacity which would optimise responses to national and regional infectious diseases outbreaks Establish meaningful engagement with donors by organising visits so they can visualise the context and the specific requirements

Abbreviations: EU, European Union; NGOs, non-governmental organisations.

###  Organisational Factors

Enablers related to organisational factors discussed in the various papers included an understanding of the regional context,^23,24^ effective information technology systems,^23^ and a functional surveillance system,^25-30^ although achieving the latter could be perceived as an aim.

Barriers included lack of understanding of the regional context (eg, presence of refugee and migrant populations),^23,31^ unstable political situations,^31,32^ a lack of political will,^33^ lack of data so a lack of awareness of the existing situation at all levels,^34,35^ including policymakers, inter-country differences, including the structure of the surveillance systems, recognition of cultural and political factors (including different languages).^36^ Others were a lack of trust and transparency, as some countries may not be inclined to report outbreaks of infectious disease (for example, as these may have potential effects on tourism and trade),^34,37^ insufficient capacity to plan, mobilise and implement control strategies,^33,38-40^ and the necessity of complying with local regulations in-country.

Two sources discussed the Asian Network for Surveillance of Resistant Pathogens (ANSORP), which was initiated in 1996.^26,41^ ANSORP is a hospital-based, non-governmental network in 14 countries (ie, Korea, China, Hong Kong, Taiwan, Japan, the Philippines, Thailand, Viet Nam, Indonesia, Malaysia, Singapore, India, Sri Lanka, and Saudi Arabia) designed to address antimicrobial resistance in the Asia Pacific region: strengthening surveillance mechanisms, organising research and collecting data to clarify the situation in each country. An ANSORP network was initiated in each participating country by experts in clinical microbiology and infectious diseases; this use of country networks helped promote understanding of the regional context. These national headquarters collaborate with the regional headquarters and a central reference laboratory in Seoul, South Korea.^26^

The Eastern Mediterranean Public Health Network (EMPHNET) was initiated as a result of regional humanitarian crises and increased likelihood of spread of infectious disease, and complex health needs among refugee and migrant populations.^23,31,42^ To promote understanding of context, focal people were identified in each country to increase collaboration through conference calls, email, and country visits, to allow the administration of effective immunisation programmes and address the potential spread of vaccine-preventable disease in the region, and to foster accountability.

The Middle East Consortium on Infectious Disease Surveillance has successfully addressed the regional context (Jordan, Israel and the Palestinian Authority) by encouraging each member country to outline their immediate objectives that then support an overall goal and work from the bottom-up, including public health staff, rather than top-down from governments or external agencies.^43^

One of the pillars of the Africa Center for Disease Control and Prevention (CDC) is to build capacity within member states of the African Union, to promote establishment of an effective surveillance system to prevent public health emergencies and reduce health inequalities.^29,30^ Strong political commitment from member states ensures that the programmes are financially feasible and sustainable, containment of potential epidemics.^28^

One barrier to successful implementation of the ANSORP network was the heterogeneity of countries involved. Some countries have much more developed surveillance and monitoring systems, and a more highly trained workforce. Guidelines and enforcement of legislation are lacking in certain countries, for example culturing specimens before prescription of antibiotics is routine in some countries and not possible currently in others. The establishment of national workgroups and guidelines is working toward increasing effective antimicrobial stewardship in what is a heterogenous region.^44^

Regional context has obvious effects on health system functioning and implementation of infectious disease control programmes. For example, EMPHNET involves countries with a significant number of asylum-seekers, forced migrants, refugees, and migrant workers. In many EMPHNET countries experiencing conflict, the health system has largely collapsed, infrastructure (eg, cold-chain facilities and suitable storage) has been destroyed, and trained healthcare personnel have been killed or displaced.^23^ Both country-specific and sub-regional (six countries) work plans aiming to strengthen routine immunisation and polio eradication programmes were developed. Data on existing gaps and requirements from each country were collected and analysed and ministries of health and country experts, with support from WHO and UNICEF, met and designed plans.^23,31^

One major issue for the ProVAC network was the lack of national data on disease burden and surveillance systems, making it difficult to plan cost-effective vaccination programmes.^34^

###  Networks

Enablers included establishing collaborative platforms,^45^ sharing knowledge,^46^ and building effective relationships, including public-private partnerships.^47^

Barriers included a lack of co-ordination between health and other sectors,^48^ lack of trust between organisations and partners, and a lack of public-private partnerships.^23,49,50^

Building effective relationships is exemplified by the African Field Epidemiology Network (AFENET), which was initiated in 2005 in collaboration with the United States (US)-CDC and was initially a collaboration between Zambia, Ghana, Uganda and Kenya.^51,52^ AFENET has a General Assembly which meets annually and is attended by representatives of the member states.^52^ A Board of Directors, in conjunction with an advisory committee of public health experts from the region, formulates policies and has general oversight of the network’s working practices. Country coordinators implement policies at country-level and report to the secretariat, which reports to the Board.

The driver for East Africa Integrated Disease Surveillance Network, established in 2000 by the East Africa Community states (ie, Kenya, Uganda, Tanzania, Rwanda, Burundi), was the incidence of cross-border malaria epidemics during the 1990s.^27^ Initiated by ministries of health and academics in the region, the main aim was to foster collaborative working to respond quickly and efficiently to cross-border disease threats, exchange and disseminate information, harmonise surveillance systems, and strengthen regional capacity.

###  Programming

Enablers included effective programme management (including incorporation of new vaccines into existing vaccination programmes),^24,53,54^ identification of priority diseases,^55^ strategy setting,^51^ and economic evaluations of programmes to identify what works best.^34^

Barriers included a lack of strategic planning and issues working across borders, with countries needing to have a sense of ownership of regional projects.^25,56^ Borders can complicate matters: the European Center for Disease Prevention and Control (ECDC) is generally not able to offer assistance or guidance to countries not in the EU or European Economic Area, which is an issue as the organisation therefore cannot respond comprehensively to infectious disease threats to the whole area.^25^

Countries in the ProVAC region have built economic evaluations into their programme planning, which has led to greater transparency and increased financial commitment from national governments.^34,57^

###  Diagnosis and Detection

Enablers included sufficient and effective laboratory capacity,^58,59^ including training staff^60,61^, and standardisation and harmonisation between laboratories across regions.^43^

Barriers included a lack of laboratory capacity and necessary equipment,^24,27^ a lack of infrastructure (eg, no healthcare facilities provided in rural areas),^24^ a lack of cold chain facilities,^53^ and a paucity of good quality data to allow planning.^27,34^

AFENET co-ordinated a laboratory capacity strengthening project that was implemented in 11 Caribbean and seven African countries, with the aim of improving laboratory quality management systems.^51^ The project supported and trained staff in rapid testing, biosafety and laboratory skills, with some staff trained through Field Epidemiology and Laboratory Training programmes (FELTP). Strategic, context-specific plans for development of laboratory quality assurance were designed to support each country, and several laboratories received accreditation.

In Europe, the ECDC has successfully strengthened epidemic preparedness by increasing countries’ capacity to detect emerging pathogens, working with national public health agencies and food safety authorities.^38^

As for many of the regions and networks discussed, lack of laboratory capacity, lack of sufficient logistics within countries to supply regional vaccine hubs, and lack of cold chain facilities are all challenges that the Partnership for Influenza Vaccine Introduction network faces.^53^ In addition, countries may not conduct routine surveillance of the same diseases or report data consistently, making data interpretation difficult.^62^

###  Human Resources

 Enablers included sufficient, appropriately qualified staff, good staff retention and effective capacity building and training.

Barriers were a lack of trained and qualified staff, particularly for microbiology, no mentorship programmes to follow-up on trained staff,^51^ high staff turnover, cultural issues (eg, ensuring that women see women healthcare providers if they so choose), and a lack of cross-disciplinary working, as staff tend to work in specialty silos.

EMPHNET hired graduates of FELTP programmes to work on implementation and sustainability of some initiatives. These interns were given a thorough grounding in organisational principles, including training on each project’s aims, policies, procedures and overall work plan.^23^ This allowed interns to work in different member countries to learn context-specific skills and knowledge while being supported and mentored by supervisors who encourage them to see the links between theory and practice.^31^ Information on programme aims and progress was sent to health-workers involved in projects, and results were disseminated as soon as they were available, fostering a sense of worth in the staff.^31^

The long-term survival and effectiveness of the AFENET FELTP programme was jeopardised as some regional governments did not have plans for continuing professional development or staff mentorship or funding in place to support these.^51^

Lack of staff retention can mean that meetings are lengthy as issues must be discussed numerous times to ensure new staff understand them, which slows implementation of activities.^27^

###  Communication

Enablers included using bulletins to keep health workers, donors, governments and the public informed of progress on, for example, vaccination programmes,^63^ dissemination of health-related publications, and having national guidelines in place. Easy communication channels between network partners is important,^64^ as is ensuring that local communities are kept aware of initiatives in their area and are able to participate as required.^65,66^ To increase the general public’s ability to access pertinent information, the EpiSouth website has a portal containing data and information about the organisation’s projects that can be accessed by anyone.^49,67^

 Barriers were preparing documents in different languages and dialects, disseminating concepts to people with little or no education, and the low literacy rate in some remote, rural regions.

Southeastern European Health Network was initiated in 2001 to enhance regional public health coordination and strengthen health systems in response to conflicts, economic issues, and humanitarian crises in the South-Eastern European countries including Bosnia, Romania and Serbia.^62^ To overcome issues with communication, regional experts worked with global organisations like WHO to design epidemiology and surveillance system training packages for communicable disease managers across the region. Countries were encouraged to perform an evaluation of their national guidelines and training curricula and helped to design context-specific training in national languages. This included adapting case definitions, harmonising procedures to meet EU standards, and preparing surveillance and pandemic preparedness plans. Fact sheets and tools were written to distribute to staff responsible for implementation.^62^

To foster effective communication, the Network for Surveillance of Pneumococcal Disease in the East African Region promotes annual supervisory visits to participating sites, to ensure standardisation of case identification, sample collection, laboratory methods, data analysis and reporting. This then feeds into intra- and inter-country meetings, which are held to discuss progress and challenges, and to pinpoint actions that need to be taken. Annual reports are presented at these meetings, the key messages of which are then written up in newsletters to be shared with all stakeholders. This style of collaboration brings different specialties and types of provider together and allows a sense of ownership at both national and local levels to develop, increasing sustainability.^24^

The Asia Partnership on Emerging Infectious Diseases Research network designs and produces policy briefs, hosts workshops with local authorities to disseminate research findings, and consults with policy makers to put into place realistic and pragmatic options, often with limited resources.^68^

###  Sustainability and Funding

Enablers included sufficient funding from governments rather than NGOs,^38^ including allocation of appropriate resources to particular programmes.^69^

 Barriers were the presence of competing priorities for funding, dependency on external rather than internal funding, difficulty securing funding for long-term programmes, including a lack of political will to provide funding for these types of programmes that might not show results for years (eg, outside of a government’s tenure), no clear planning for how to allocate and use available funding, and underfunded healthcare systems where infectious disease prevention is not a priority.

The Mekong Basin Disease Surveillance aims to facilitate cross-border disease outbreak investigations by sharing surveillance data. However, this has been complicated by the reluctance of some member countries to share data and the necessity of long-term support from development partners. The network has highlighted the importance of political support from regional governments with regard to funding, and the central role of staff training and capacity building to promote sustainability.^33^

A key constraint to the successful working of the South African Center for Infectious Disease Surveillance is the lack of adequate, consistent and secure funding to finance initiatives like the filovirus surveillance system and regional conferences where stakeholders can meet and discuss potential projects.^70^

## Discussion

###  Key Findings 

This review provided an overview of the scope and main findings of peer-reviewed and non-peer-reviewed literature on the initiation and operationalisation of regional infectious disease control bodies. There was a clear disparity in numbers of articles focused on different regions. For example, we identified 14 relevant sources that mainly discussed initiatives in Europe compared with eight focused on the Eastern Mediterranean region and five specifically centred on SEA. The latter two regions have hosted major infectious disease outbreaks such as the SARS-coronavirus 1 (CoV1) outbreak in China in 2002 and the emergence of Middle East respiratory syndrome in Saudi Arabia in 2012.^71^

Most of these regional bodies and networks were established with the aim of enhancing surveillance and building the outbreak investigation capacity of technical teams and laboratories. Three US-CDC funded bodies were established in 2009, when, among other drivers, there was a growing fear that the humanitarian crisis in West Asia could contribute to the spread of infectious diseases to Europe and other high-income regions. These bodies aimed to build effective surveillance and immunisation systems in conflict-affected regions to help public health systems eradicate diseases such as polio. Other networks aimed to build regional infectious disease research capacity, while focusing on improving laboratory quality management systems and meeting international standards.^23,31,42^ One example is RESAOLAB, a clinical laboratory network designed to strengthen disease surveillance in west Africa, which develops and implements training programmes in countries in the region.^72^

Some key achievements of these collaborative networks were increasing trust between member states,^73^ building capacities of laboratory and technical staff, and creating new channels for exchanging expertise among different stakeholders within member states (eg, South-East Asia Infectious Disease Clinical Research Network [SEAICRN], EpiSouth Network, EMPHNET).^23,49,50^ Additionally, these bodies helped strengthen preparedness and accelerate responses to outbreaks in their regions, such as influenza in the Eastern Mediterranean region and dengue fever in Europe.^43,74^ While this is a key metric, it is difficult to ascertain what (or even whether) specific networks contributed to positive and effective outbreak responses. This underlines the importance of inbuilt monitoring and evaluation from the beginning.

Monitoring the performance of implemented programmes, including the effectiveness of training for technical staff, is a key challenge for regional networks, and, again, measuring this kind of success is not simple. One source suggested implementation of a regional peer audit mechanism, which could be an effective tool for facilitating cross-country learning and enhancing the performance of staff in the region.^75^

Conflict and political instability were identified as the most significant challenges to establishing regional collaborations.^31^ Another is the sustainability of funding, which may be improved by visits from external donors to regional staff, encouraging feedback on activities and responding to staff concerns. This type of visit gives donors and other stakeholders a context-specific perspective from staff embedded within regional and national realities. This perspective is essential for the longevity and success of any project, and to inform future planning and implementation activities.^23^ Lack of capacity of infectious disease laboratories and technical staff were discussed as major challenges. The experiences of SEAICRN in building the capacity of infectious disease laboratories in the region are illustrative. SEAICRN played a positive role in speeding responses to the spread of drug-resistant influenza A and bird flu outbreaks and facilitated development of a clinical research protocol in Viet Nam. This was achieved by assessing the capacity of hospital clinical laboratories against international standards, employing a qualified assessor to identify performance issues in selected laboratories, and establishing a laboratory networking system to include regular meetings and encourage the exchange of experiences and ideas among laboratory staff from different countries.^50^

###  Implications 

Based on lessons from existing regional bodies and networks, we therefore consider that necessary steps for successful initiation of an ASEAN infectious disease control body should include: (*i*) as many interested stakeholders as possible from the beginning - including community representatives if possible - sharing an open forum and discussing ideas, aims, opportunities, barriers, and ways of working; (*ii*) ensuring that contextual factors, eg, potential disease drivers, political-economy, socio-cultural, linguistic, geographical, and resources are considered; and (*iii*) ensuring that staff feel valued, through capacity-building and mentoring programmes, to reduce turnover and promote stability and sustainability in the organisational structure. Contextual factors are key to developing an effective organisation in a specific region. Different contexts may affect, for example, whether political will for cooperation and change exists at national levels and specific models that have worked in other regions should not be transplanted without adaptation to the ASEAN region. Thus, it is essential for a diverse and interdisciplinary group of stakeholders, with regional disease control knowledge and experience, to be involved from inception.

It is important to set clear objectives and realistic governance structures for regional collaboration, where the member states can delineate their national goals and expectations from this network, and agree how to mainstream these within the overarching regional goals. The use of a modified Delphi approach, which is a prioritisation exercise using a questionnaire, helped in reaching a consensus on priority areas for the development of a collaborative body in Europe to tackle infectious diseases.^76^

Initiation of AFENET points up the importance of including a clear organisational structure from the beginning, with a Board of Directors, working with an advisory committee of public health experts from the region, to formulate policies and oversee the network’s working practices. On the ground national coordinators implement policies and report any issues to the general secretariat, which feeds back to the Board, which in turn advises and directs.^51,52^

###  Limitations 

 This review had several limitations. Most sources were in English, and although unlikely as publishing trends favour English and we included all publication languages with an English abstract, some relevant studies published in other languages may have been overlooked. We may have missed some studies not indexed in the databases we searched. We did not critically appraise source quality as scoping reviews are designed to identify and synthesise lessons learned from the existing research, and methodological appraisal could have eliminated useful sources. Additionally, the heterogeneity of the studies included precluded a comprehensive and useful quality appraisal.

## Conclusion

 Operationalisation of regional infectious disease bodies is complicated, but with extensive groundwork, and focus on organisational factors, diagnosis and detection, human resources, communication, accreditation, funding, and sustainability, it is achievable. Ways to promote success are to include as many stakeholders as possible from the initiation stages, to ensure that context-specific factors are considered, and to encourage employees through capacity-building and mentoring to ensure they feel valued and reduce staff turnover.

## Ethical issues

 Not applicable.

## Competing interests

 Authors declare that they have no competing interests.

## Authors’ contributions

 NH conceived the study. ADB led the writing of the manuscript. All authors were involved in the data acquisition, data analysis and interpretation, and contributed to critical content and to drafting the manuscript.

## Funding

 This work was supported by Health Systems Research Institute, Thailand.

## Supplementary files

Supplementary file 1. Sources Included in Analysis.
