# Supplementary material for: Operationalising Regional Cooperation for Infectious Disease Control: A Scoping Review of Regional Disease Control Bodies and Networks
Source: Int J Health Policy Manag. 2021 Dec 26;11(11):2392–403. doi: 10.34172/ijhpm.2021.176 (PMC9818116; doi:10.34172/ijhpm.2021.176)
Supplement: Supplementary file 1 — Sources Included in Analysis. [file ijhpm-11-2392-s001.pdf]

**Article title:** Operationalising Regional Cooperation for Infectious Disease Control: A Scoping Review of Regional Disease Control Bodies and Networks

**Journal name:** International Journal of Health Policy and Management

**Authors' information:** Anna Durrance-Bagale[1,2]*, Manar Marzouk[1], Sunanda Agarwal[3], Aparna Ananthakrishnan[4], Sarah Gan[1], Michiko Hayashi[1], Beth Jacob-Chow[1], Koh Jiayun[1], Lam Sze Tung[1], Hala Mkhallalati1, Sanjida Newaz[5], Maryam Omar[6], Manit Sittimart[4], Mengieng Ung[7], Yang Yuze[1], Hsu Li Yang[1], Natasha Howard[1,2]

[1]National University of Singapore, Saw Swee Hock School of Public Health, Singapore, Singapore.

[2]London School of Hygiene and Tropical Medicine, London, UK.

[3]Stanford Distinguished Careers Institute, Stanford, CA, USA.

[4]Health Intervention and Technology Assessment Program, Ministry of Public Health, Nonthaburi, Thailand.

[5]Department of Community Health Sciences, Rady Faculty of Health Sciences, University of Manitoba, Winnipeg, MB, Canada.

[6]Chelsea and Westminster Hospital NHS Foundation Trust, London, UK.

[7]Lee Kuan Yew Centre for Innovative Cities, Singapore University of Technology and Design, Singapore, Singapore.

(*Corresponding author: anna.durrance-bagale@lshtm.ac.uk)

**Supplementary file 1.** Sources Included in Analysis

**Table 1. Sources included in analysis**

| First author (year) | Region | Organisation/body/network name | References |
|---|---|---|---|
| Al Gunaid (2019) | Asia | EMPHNET | 23 |
| Al Nsour 2019) | Asia | EMPHNET | 31 |
| Al Nsour (2020) | Asia | EMPHNET | 42 |
| Albiger (2018) | Europe | ECDC | 38 |
| Amos (2009) | Africa | netSPEAR | 24 |
| Anderson (2020) | Europe | ECDC | 25 |
| Bell (2018) | Western Pacific | PIVI | 53 |
| Bino (2013) | Europe | SEEHN | 62 |
| Birmingham (1997) | Africa | EPI | 35 |

| | | | |
|---|---|---|---|
| Blau (2015) | Europe | ProVAC | 34 |
| Chung (2013) | Asia | ANSORP | 26 |
| Cuenca-Estrella (2008) | Americas | Regional Network for the Surveillance of Invasive Fungal Infections and Susceptibility to Antifungals | 22 |
| Dente (2009) | Europe | Epi-South | 67 |
| Dente (2018) | Europe | MediLab Secure | 48 |
| Dente (2017) | Europe | Epi-South | 49 |
| Hoff (2011) | Asia | REDI | 58 |
| Ison (2012) | Europe | Euro-GASP | 60 |
| Jourdan-Hidalgo (1999) | Americas | AMPES | 21 |
| Kabugo (2018) | Africa/Americas | AFENET | 51 |
| Kim (2011) | Americas | ARICABA | 36 |
| Kimball (2008) | Africa/Asia | MECIDS/MBDS | 73 |
| Lee (2021) | Asia | ANSORP | 41 |
| LeFrancois (2010) | Americas | CaribVET | 44 |
| Leventhal (2013) | Asia | MECIDS | 43 |
| Malik (2020) | Asia | WHO Eastern Mediterranean Region Network | 32 |
| Malik (2013) | Asia | WHO Eastern Mediterranean Region Network | 37 |
| McKendrick (2005) | Europe | EUMS | 61 |
| Mukanga (2011) | Africa | AFENET | 52 |
| Mwenda (2017) | Africa | ARSN | 54 |
| Najjar-Pellet (2013) | Africa | RESAOLAB | 72 |
| Nkengasong (2017) | Africa | Africa CDC | 30 |
| Noormal (2019) | Asia | EMPHNET | 46 |
| Oboh (2020) | Africa | Africa CDC | 28 |
| Ogutu (2010) | Africa | MCTA | 59 |
| Olveda (2010) | Asia | RNAS | 63 |
| Ope (2013) | Africa | EAIDSNet | 27 |
| Phommasack (2013) | Asia | MBDS | 33 |
| Pinell-McNamara (2017) | Americas | LAPP | 64 |
| Postigo (2010) | Asia | WHO Eastern Mediterranean Region Network | 69 |
| Ravishankar (2015) | Africa | EAPHLNP | 47 |

| | | | |
|---|---|---|---|
| Rweyemamu (2013) | Africa | SACIDS | 70 |
| Schneidman (2018) | Africa | EAPHLNP | 75 |
| Silkavute (2013) | Asia | APEIR | 68 |
| Solet (2014) | Africa | SEGA | 19 |
| Souares (2000) | Pacific | PPHSN | 56 |
| Sprenger (1998) | Europe | EU | 20 |
| Torres (2017) | Americas | LACER | 65 |
| Toscano (2013) | Americas | ProVAC | 57 |
| VanLoock (2002) | Europe | ECDC | 39 |
| VanLoock (2001) | Europe | Network Committee for Epidemiological Surveillance and Control of Communicable Diseases | 40 |
| Varan (2015) | All regions | ASEAN; BIDS; COMISCA; ICS; EAIDSNet; ECDC; MBDS; MECIDS; PPHSN; SACIDS; SEEHN; UNASUR | 55 |
| Varma (2020) | Africa | Africa CDC | 29 |
| Vung (2011) | Asia | MBDS | 66 |
| Weinberg (1999) | Europe | EU Charter Group | 76 |
| Wertheim (2010) | Asia | SEAICRN | 50 |
| Wilder-Smith (2019) | Americas | ZikaPLAN | 45 |
| Zeller (2013) | Europe | ECDC | 74 |

AFENET: African Field Epidemiology Network; AMPES: American Regional Planning, Programming, Monitoring, and Evaluation System; ANSORP: Asian Network for Surveillance of Resistant Pathogens; APEIR: Asia Partnership on Emerging Infectious Diseases; ARSN: African Rotavirus Surveillance Network; ASEAN: Association of Southeast Asian Networks; ARICABA: Surveillance Network System for Emerging Infectious Diseases in the Caribbean; BIDS: Border Infectious Disease Surveillance; CDC: Center for Disease Control and Prevention; COMISCA: Council of Ministers of Health of Central America and the Dominican Republic; EAIDSNet: East African Integrated Disease Surveillance Network; EAPHLNP: East Africa Public Health Laboratory Networking Project; ECDC: European Center for Disease Prevention and Control; EMPHNET: Eastern Mediterranean Public Health Network; EPI: Expanded Program on Immunization; EU: European Union; EUMS: European union of Medical Specialties; Euro-GASP: European Gonococcal Antimicrobial Susceptibility Programme; ICS: International Circumpolar Surveillance; LACER: Latin American Coalition for Escherichia Coli Research; LAPP: Latin American Pertussis Project; MBDS: Mekong Basin Disease Surveillance; MCTA: Malaria Clinical Trial Alliance; MECIDS: Middle East Consortium on Infectious Disease Surveillance; netSPEAR: Network for Surveillance of Pneumococcal Disease in the East African Region; PIVI: Partnership for Influenza Vaccine Introduction; PPHSN: Pacific Public Health Surveillance Network; REDI: Regional Emerging Diseases Intervention; RESAOLAB: West African Network of Biomedical Analysis Laboratories; RNAS: Regional Network on Asian Schistosomiasis and Other Helminth Zoonoses; SACIDS: Southern African Center for Infectious Disease Surveillance; SEAICRN: South East Asia Infectious Disease Clinical Research Network; SEEHN: Southeast European Health Network; SEGA: Surveillance des épidémies et gestion des alertes; UNASUR: Union of South American Nations.
